# Title: Risk factors of prolonged corrected QT interval among patients with mental disorders

**DOI:** 10.1192/j.eurpsy.2021.1285

**Published:** 2021-08-13

**Authors:** N. Smaoui, W. Abid, I. Lajmi, S. Omri, R. Feki, M. Maalej Bouali, N. Charfi, N. Zouari, J. Ben Thabet, L. Zouari, M. Maalej

**Affiliations:** Psychiatry C Department, Hedi chaker University hospital, sfax, Tunisia

**Keywords:** Mental disorders, Antipsychotic drugs, Risk factors, Prolonged qt interval

## Abstract

**Introduction:**

There is an increased rate of sudden cardiac death in mental health patients. Studies provide consistent evidence that prolonged QT interval is associated with higher risk of all-cause and cardiovascular mortality.

**Objectives:**

This study aimed to assess the prevalence of prolonged QTc interval (corrected QT>450 milliseconds) and to determine the possible factors in hospitalized psychiatric patients.

**Methods:**

We reviewed records of all mental health inpatient admissions to the psychiatry “C” department at Hedi Chaker university hospital in Sfax, between 1 february and 30 april 2019. Electrocardiogram (ECG) availability was noted and QTc interval was manually measured. Sociodemographic, clinical, biological and therapeutic data were collected.

**Results:**

Of 68 mental health inpatient admissions, 59 (86.6 %) presentations had an ECG. A total of seven (11.8 %) had a prolonged QTc interval. These seven patients were treated with typical antipsychotics. Of the 7 patients with a prolonged QTc, 4 patients (57.1%) suffered from schizophrenia. QTc prolongation was significantly correlated with the presence of a recent physical trauma (p = 0.021), dietary restriction (p = 0.026), and taking at least two antipsychotics (p = 0.008). Moreover, this prolongation of QTc was linked to a longer duration of disease and an older age, without significant associations.

**Conclusions:**

Our study supports an association between a prolonged QTc interval and clinical situations at risk and antipsychotic polypharmacy. However, a larger study with routine ECG screening is required to better assess the significance of this problem.

